# 2,3-Diamino­pyridinium 4-meth­oxy­quinoline-2-carboxyl­ate

**DOI:** 10.1107/S1600536812047642

**Published:** 2012-11-24

**Authors:** Kaliyaperumal Thanigaimani, Nuridayanti Che Khalib, Suhana Arshad, Ibrahim Abdul Razak

**Affiliations:** a School of Physics, Universiti Sains Malaysia, 11800 USM, Penang, Malaysia

## Abstract

In the 4-meth­oxy­quinoline-2-carboxyl­ate anion of the title salt, C_5_H_8_N_3_^+^·C_11_H_8_NO_3_^−^, the dihedral angle between the quinoline ring system and the carboxyl­ate group is 16.54 (15)°. In the crystal, the cations and anions are linked *via* N—H⋯O and N—H⋯N hydrogen bonds, forming a centrosymmetric 2 + 2 aggregate with *R*_2_^2^(9) and *R*_4_^2^(8) ring motifs. These units are further connected *via* N—H⋯O hydrogen bonds into a layer parallel to the *bc* plane. The crystal structure is also stabilized by weak C—H⋯O hydrogen bonds and π–π inter­actions between pyridine rings [centroid–centroid distance = 3.5886 (8) Å] and between pyridine and benzene rings [centroid–centroid distance = 3.6328 (8) Å].

## Related literature

For background to the chemistry of substituted pyridines, see: Pozharski *et al.* (1997 ▶); Katritzky *et al.* (1996 ▶). For background to and the biological activity of quinoline derivatives, see: Morimoto *et al.* (1991 ▶); Markees *et al.* (1970 ▶). For a related structure, see: Hemamalini & Fun (2011 ▶). For hydrogen-bond motifs, see: Bernstein *et al.* (1995 ▶). For bond-length data, see: Allen *et al.* (1987 ▶). For the stability of the temperature controller used for the data collection, see: Cosier & Glazer (1986 ▶).
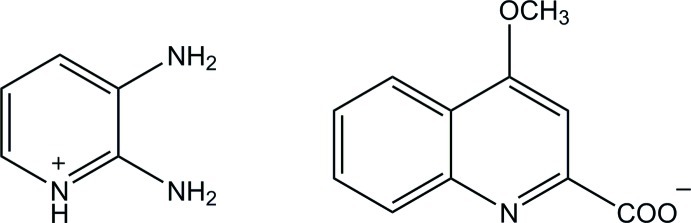


## Experimental

### 

#### Crystal data

C_5_H_8_N_3_^+^·C_11_H_8_NO_3_^−^
*M* *_r_* = 312.33
Monoclinic, 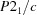

*a* = 12.4338 (12) Å
*b* = 7.7462 (7) Å
*c* = 19.4626 (14) Å
β = 128.806 (4)°
*V* = 1460.8 (2) Å^3^
*Z* = 4
Mo *K*α radiation
μ = 0.10 mm^−1^
*T* = 100 K
0.24 × 0.21 × 0.11 mm

#### Data collection

Bruker SMART APEXII CCD area-detector diffractometer
Absorption correction: multi-scan (*SADABS*; Bruker, 2009 ▶) *T*_min_ = 0.976, *T*_max_ = 0.989
17919 measured reflections
4820 independent reflections
3806 reflections with *I* > 2σ(*I*)
*R*_int_ = 0.036

#### Refinement

*R*[*F* ^2^ > 2σ(*F* ^2^)] = 0.047
*wR*(*F* ^2^) = 0.131
*S* = 1.02
4820 reflections
229 parameters
H atoms treated by a mixture of independent and constrained refinement
Δρ_max_ = 0.54 e Å^−3^
Δρ_min_ = −0.24 e Å^−3^

### 

Data collection: *APEX2* (Bruker, 2009 ▶); cell refinement: *SAINT* (Bruker, 2009 ▶); data reduction: *SAINT*; program(s) used to solve structure: *SHELXTL* (Sheldrick, 2008 ▶); program(s) used to refine structure: *SHELXTL*; molecular graphics: *SHELXTL*; software used to prepare material for publication: *SHELXTL* and *PLATON* (Spek, 2009 ▶).

## Supplementary Material

Crystal structure: contains datablock(s) global, I. DOI: 10.1107/S1600536812047642/is5215sup1.cif

Structure factors: contains datablock(s) I. DOI: 10.1107/S1600536812047642/is5215Isup2.hkl

Supplementary material file. DOI: 10.1107/S1600536812047642/is5215Isup3.cml

Additional supplementary materials:  crystallographic information; 3D view; checkCIF report

## Figures and Tables

**Table 1:** Hydrogen-bond geometry (Å, °)

*D*—H⋯*A*	*D*—H	H⋯*A*	*D*⋯*A*	*D*—H⋯*A*
N3—H2⋯O3	0.91 (2)	1.890 (19)	2.7670 (18)	162.8 (19)
N2—H3⋯N1	0.94 (2)	1.94 (3)	2.843 (2)	162.5 (19)
N3—H1⋯O3^i^	0.89 (2)	1.94 (2)	2.812 (2)	163.4 (16)
N4—H4⋯O2^ii^	0.893 (18)	1.978 (19)	2.8617 (15)	169.8 (17)
N4—H5⋯O2^i^	0.88 (2)	2.17 (3)	2.9419 (18)	146 (3)
C4—H4*A*⋯O2^iii^	0.95	2.45	3.3529 (15)	158

Symmetry codes: (i) 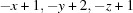; (ii) 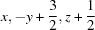; (iii) 
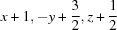
.

**Fig. 1.**
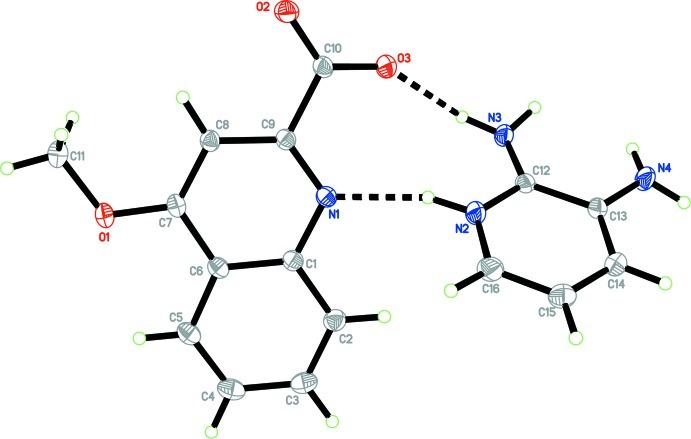
The molecular structure of the title compound with atom labels with 50% probability displacement ellipsoids.

**Fig. 2.**
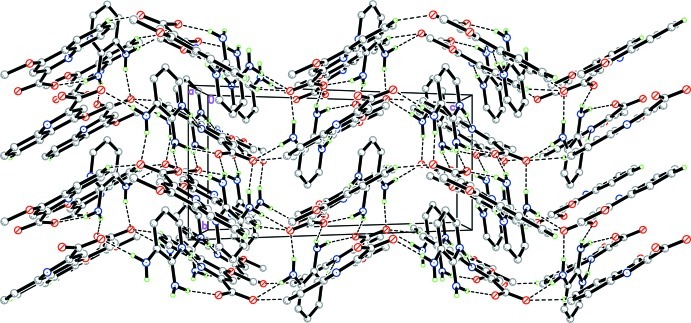
The crystal packing of the title compound. The H atoms not involved in the intermolecular interactions (dashed lines) have been omitted for clarity.

## supplementary crystallographic information

### Comment

Pyridine and its derivatives play an important role in heterocyclic chemistry (Pozharski *et al.*, 1997; Katritzky *et al.*, 1996). Quinolines and their derivatives are very important compounds because of their wide occurrence in natural products (Morimoto *et al.*, 1991) and biologically active compounds (Markees *et al.*, 1970). Recently, hydrogen-bonding patterns involving 4-methoxyquinolinium-2-carboxylate dihydrate (Hemamalini & Fun, 2011) have been reported. In order to study some interesting hydrogen bonding interactions, the synthesis and structure of the title compound, (I), is presented here.

The asymmetric unit (Fig. 1) contains one 2,3-diaminopyridinium cation and one 4-methoxyquinoline-2-carboxylate anion. In the 2,3-diaminopyridinium cation, the protonated N2 atom has lead to a slight increase in the C12—N2—C16 angle to 123.67 (14)°. The 2,3-diaminopyridinium cation is planar, with a maximum deviation of 0.005 (1) Å for atom C14. The bond lengths (Allen *et al.*, 1987) and angles are normal.

In the crystal packing (Fig. 2), the 2-amino groups (N3 and N4) are hydrogen bonded to the carboxylate oxygen atoms (O3 and O2) *via* a pair of intermolecular N3—H1···O3^i^ and N4—H5···O2^i^ hydrogen bonds (symmetry code in Table 1), forming an *R*_2_^2^(9) (Bernstein *et al.*, 1995) ring motif. These motifs are crosslinked *via* N3—H2···O3 and N2—H3···N1 hydrogen bonds to produce a DDAA array (where D is a hydrogen-bond donor and A is a hydrogen-bond acceptor) with *R*_2_^2^(9) and *R*_4_^2^(8) motifs. The crystal structure also features weak N4—H4···O2^ii^ and C4—H4A···O2^iii^ hydrogen bonds (symmetry codes in Table 1). Furthermore, the crystal structure is stabilized by the following π–π interactions: (*a*) between pyridine rings (N2/C12–C16, centroid *Cg*4) *Cg*4···*Cg*4 (1 - *x*, 1 - *y*, 1 - *z*) 3.5886 (8) Å and (*b*) between pyridine (N1/C1/C6–C9, centroid *Cg*1) and benzene (C1–C6, centroid *Cg*2) rings *Cg*1···*Cg*2 (2 - *x*, 1 - *y*, 1 - *z*) 3.6328 (8) Å.

### Experimental

Hot methanol solutions (20 ml) of 2,3-diaminopyrimidine (27 mg, Aldrich) and 4-Methoxy-2-quinolinecarboxylic acid (50 mg, Merck) were mixed and warmed over a heating magnetic stirrer hotplate for a few minutes. The resulting solution was allowed to cool slowly at room temperature and crystals of the title compound (I) appeared after a few days.

### Refinement

N-bound H Atoms were located in a difference Fourier maps and refined freely [N—H = 0.88 (2)–0.936 (18) Å]. The remaining hydrogen atoms were positioned (C—H = 0.95 and 0.98 Å) and refined using a riding model, with *U*_iso_(H) = 1.2*U*_eq_(C) or 1.5*U*_eq_(methyl C). A rotating-group model was used for the methyl group.

### Crystal data

**Table:**

C_5_H_8_N_3_^+^·C_11_H_8_NO_3_^−^	*F*(000) = 656
*M**_r_* = 312.33	*D*_x_ = 1.420 Mg m^−^^3^
Monoclinic, *P*2_1_/*c*	Mo *K*α radiation, λ = 0.71073 Å
Hall symbol: -P 2ybc	Cell parameters from 4575 reflections
*a* = 12.4338 (12) Å	θ = 2.7–31.3°
*b* = 7.7462 (7) Å	µ = 0.10 mm^−^^1^
*c* = 19.4626 (14) Å	*T* = 100 K
β = 128.806 (4)°	Block, brown
*V* = 1460.8 (2) Å^3^	0.24 × 0.21 × 0.11 mm
*Z* = 4	

### Data collection

**Table:**

Bruker SMART APEXII CCD area-detector diffractometer	4820 independent reflections
Radiation source: fine-focus sealed tube	3806 reflections with *I* > 2σ(*I*)
Graphite monochromator	*R*_int_ = 0.036
φ and ω scans	θ_max_ = 31.4°, θ_min_ = 2.1°
Absorption correction: multi-scan (*SADABS*; Bruker, 2009)	*h* = −18→18
*T*_min_ = 0.976, *T*_max_ = 0.989	*k* = −11→11
17919 measured reflections	*l* = −28→28

### Refinement

**Table:**

Refinement on *F*^2^	Primary atom site location: structure-invariant direct methods
Least-squares matrix: full	Secondary atom site location: difference Fourier map
*R*[*F*^2^ > 2σ(*F*^2^)] = 0.047	Hydrogen site location: inferred from neighbouring sites
*wR*(*F*^2^) = 0.131	H atoms treated by a mixture of independent and constrained refinement
*S* = 1.02	*w* = 1/[σ^2^(*F*_o_^2^) + (0.0667*P*)^2^ + 0.4735*P*] where *P* = (*F*_o_^2^ + 2*F*_c_^2^)/3
4820 reflections	(Δ/σ)_max_ < 0.001
229 parameters	Δρ_max_ = 0.54 e Å^−^^3^
0 restraints	Δρ_min_ = −0.24 e Å^−^^3^

### Special details

**Table:**

Experimental. The crystal was placed in the cold stream of an Oxford Cryosystems Cobra open-flow nitrogen cryostat (Cosier & Glazer, 1986) operating at 100.0 (1) K.
Geometry. All e.s.d.'s (except the e.s.d. in the dihedral angle between two l.s. planes) are estimated using the full covariance matrix. The cell e.s.d.'s are taken into account individually in the estimation of e.s.d.'s in distances, angles and torsion angles; correlations between e.s.d.'s in cell parameters are only used when they are defined by crystal symmetry. An approximate (isotropic) treatment of cell e.s.d.'s is used for estimating e.s.d.'s involving l.s. planes.
Refinement. Refinement of *F*^2^ against ALL reflections. The weighted *R*-factor *wR* and goodness of fit *S* are based on *F*^2^, conventional *R*-factors *R* are based on *F*, with *F* set to zero for negative *F*^2^. The threshold expression of *F*^2^ > σ(*F*^2^) is used only for calculating *R*-factors(gt) *etc*. and is not relevant to the choice of reflections for refinement. *R*-factors based on *F*^2^ are statistically about twice as large as those based on *F*, and *R*- factors based on ALL data will be even larger.

### Fractional atomic coordinates and isotropic or equivalent isotropic displacement parameters (Å^2^)

**Table:**

	*x*	*y*	*z*	*U*_iso_*/*U*_eq_	
O1	1.05026 (9)	0.82361 (12)	0.41897 (6)	0.02018 (19)	
O2	0.57301 (9)	0.98701 (12)	0.31851 (6)	0.02111 (19)	
O3	0.59315 (10)	0.93999 (14)	0.43875 (7)	0.0273 (2)	
N1	0.82921 (10)	0.75610 (13)	0.51232 (6)	0.0165 (2)	
C1	0.96096 (11)	0.69323 (14)	0.55691 (7)	0.0157 (2)	
C2	1.02304 (12)	0.60542 (16)	0.63772 (8)	0.0195 (2)	
H2A	0.9720	0.5869	0.6583	0.023*	
C3	1.15663 (13)	0.54699 (16)	0.68650 (8)	0.0214 (2)	
H3A	1.1969	0.4874	0.7403	0.026*	
C4	1.23432 (12)	0.57458 (16)	0.65750 (8)	0.0222 (2)	
H4A	1.3269	0.5347	0.6921	0.027*	
C5	1.17713 (12)	0.65876 (16)	0.57956 (8)	0.0193 (2)	
H5A	1.2303	0.6772	0.5605	0.023*	
C6	1.03897 (11)	0.71840 (15)	0.52728 (7)	0.0159 (2)	
C7	0.97352 (11)	0.80680 (15)	0.44581 (8)	0.0156 (2)	
C8	0.84151 (11)	0.87022 (15)	0.40172 (7)	0.0156 (2)	
H8A	0.7961	0.9300	0.3474	0.019*	
C9	0.77543 (11)	0.84423 (15)	0.43910 (7)	0.0151 (2)	
C10	0.63500 (12)	0.92902 (15)	0.39471 (8)	0.0167 (2)	
C11	0.98637 (14)	0.90182 (19)	0.33460 (9)	0.0239 (3)	
H11A	1.0535	0.9092	0.3239	0.036*	
H11B	0.9545	1.0180	0.3339	0.036*	
H11C	0.9075	0.8316	0.2884	0.036*	
N2	0.69022 (11)	0.60036 (14)	0.56941 (7)	0.0186 (2)	
N3	0.61802 (11)	0.86007 (14)	0.58672 (7)	0.0187 (2)	
N4	0.53416 (12)	0.68143 (16)	0.67353 (8)	0.0242 (2)	
C12	0.63244 (11)	0.68819 (15)	0.59866 (7)	0.0156 (2)	
C13	0.59474 (11)	0.59571 (16)	0.64456 (7)	0.0169 (2)	
C14	0.62055 (13)	0.41992 (16)	0.65640 (8)	0.0205 (2)	
H14A	0.5978	0.3557	0.6873	0.025*	
C15	0.67973 (13)	0.33474 (17)	0.62363 (9)	0.0230 (3)	
H15A	0.6954	0.2138	0.6316	0.028*	
C16	0.71448 (13)	0.42631 (16)	0.58045 (8)	0.0222 (2)	
H16A	0.7550	0.3702	0.5583	0.027*	
H1	0.5578 (18)	0.916 (2)	0.5893 (11)	0.026 (4)*	
H2	0.6256 (18)	0.899 (2)	0.5461 (11)	0.025 (4)*	
H3	0.7185 (18)	0.663 (2)	0.5421 (12)	0.032 (5)*	
H4	0.5352 (19)	0.630 (2)	0.7151 (12)	0.032 (5)*	
H5	0.525 (2)	0.795 (3)	0.6708 (13)	0.041 (5)*	

### Atomic displacement parameters (Å^2^)

**Table:**

	*U*^11^	*U*^22^	*U*^33^	*U*^12^	*U*^13^	*U*^23^
O1	0.0191 (4)	0.0250 (4)	0.0228 (4)	0.0005 (3)	0.0162 (4)	0.0014 (3)
O2	0.0206 (4)	0.0255 (5)	0.0171 (4)	0.0057 (3)	0.0118 (3)	0.0008 (3)
O3	0.0298 (5)	0.0345 (5)	0.0303 (5)	0.0146 (4)	0.0251 (4)	0.0128 (4)
N1	0.0184 (4)	0.0158 (4)	0.0181 (4)	0.0026 (3)	0.0128 (4)	0.0007 (3)
C1	0.0179 (5)	0.0139 (5)	0.0172 (5)	0.0012 (4)	0.0119 (4)	−0.0007 (4)
C2	0.0222 (5)	0.0188 (5)	0.0184 (5)	0.0021 (4)	0.0132 (5)	0.0010 (4)
C3	0.0217 (5)	0.0193 (6)	0.0169 (5)	0.0013 (4)	0.0090 (4)	0.0009 (4)
C4	0.0161 (5)	0.0196 (6)	0.0229 (6)	0.0010 (4)	0.0083 (5)	−0.0010 (4)
C5	0.0158 (5)	0.0183 (5)	0.0226 (6)	−0.0006 (4)	0.0114 (4)	−0.0026 (4)
C6	0.0159 (5)	0.0139 (5)	0.0180 (5)	−0.0003 (4)	0.0107 (4)	−0.0024 (4)
C7	0.0167 (5)	0.0151 (5)	0.0189 (5)	−0.0021 (4)	0.0130 (4)	−0.0028 (4)
C8	0.0174 (5)	0.0151 (5)	0.0165 (5)	0.0003 (4)	0.0117 (4)	−0.0007 (4)
C9	0.0171 (5)	0.0142 (5)	0.0165 (5)	0.0013 (4)	0.0117 (4)	−0.0009 (4)
C10	0.0182 (5)	0.0155 (5)	0.0197 (5)	0.0020 (4)	0.0136 (4)	0.0005 (4)
C11	0.0258 (6)	0.0296 (7)	0.0236 (6)	0.0015 (5)	0.0191 (5)	0.0027 (5)
N2	0.0216 (5)	0.0197 (5)	0.0207 (5)	0.0027 (4)	0.0162 (4)	0.0017 (4)
N3	0.0231 (5)	0.0176 (5)	0.0226 (5)	0.0035 (4)	0.0178 (4)	0.0037 (4)
N4	0.0329 (6)	0.0248 (6)	0.0300 (6)	0.0098 (4)	0.0271 (5)	0.0095 (4)
C12	0.0149 (4)	0.0183 (5)	0.0146 (5)	0.0021 (4)	0.0097 (4)	0.0010 (4)
C13	0.0158 (5)	0.0205 (5)	0.0159 (5)	0.0015 (4)	0.0107 (4)	0.0023 (4)
C14	0.0218 (5)	0.0195 (5)	0.0231 (6)	0.0013 (4)	0.0154 (5)	0.0038 (4)
C15	0.0254 (6)	0.0174 (5)	0.0260 (6)	0.0029 (4)	0.0160 (5)	0.0017 (5)
C16	0.0263 (6)	0.0197 (6)	0.0237 (6)	0.0049 (4)	0.0172 (5)	0.0004 (5)

### Geometric parameters (Å, º)

**Table:**

O1—C7	1.3509 (13)	C9—C10	1.5284 (15)
O1—C11	1.4351 (15)	C11—H11A	0.9800
O2—C10	1.2501 (14)	C11—H11B	0.9800
O3—C10	1.2537 (14)	C11—H11C	0.9800
N1—C9	1.3227 (15)	N2—C12	1.3475 (15)
N1—C1	1.3755 (14)	N2—C16	1.3688 (16)
C1—C2	1.4179 (16)	N2—H3	0.936 (18)
C1—C6	1.4185 (16)	N3—C12	1.3441 (15)
C2—C3	1.3743 (17)	N3—H1	0.893 (18)
C2—H2A	0.9500	N3—H2	0.905 (17)
C3—C4	1.4073 (19)	N4—C13	1.3634 (16)
C3—H3A	0.9500	N4—H4	0.893 (18)
C4—C5	1.3712 (18)	N4—H5	0.88 (2)
C4—H4A	0.9500	C12—C13	1.4337 (16)
C5—C6	1.4174 (15)	C13—C14	1.3848 (17)
C5—H5A	0.9500	C14—C15	1.4041 (18)
C6—C7	1.4242 (16)	C14—H14A	0.9500
C7—C8	1.3808 (15)	C15—C16	1.3606 (19)
C8—C9	1.4121 (16)	C15—H15A	0.9500
C8—H8A	0.9500	C16—H16A	0.9500
			
C7—O1—C11	117.67 (9)	O3—C10—C9	117.34 (10)
C9—N1—C1	117.42 (10)	O1—C11—H11A	109.5
N1—C1—C2	118.35 (10)	O1—C11—H11B	109.5
N1—C1—C6	122.84 (10)	H11A—C11—H11B	109.5
C2—C1—C6	118.76 (10)	O1—C11—H11C	109.5
C3—C2—C1	120.42 (11)	H11A—C11—H11C	109.5
C3—C2—H2A	119.8	H11B—C11—H11C	109.5
C1—C2—H2A	119.8	C12—N2—C16	123.66 (11)
C2—C3—C4	120.61 (11)	C12—N2—H3	117.9 (11)
C2—C3—H3A	119.7	C16—N2—H3	118.4 (11)
C4—C3—H3A	119.7	C12—N3—H1	119.7 (11)
C5—C4—C3	120.36 (11)	C12—N3—H2	113.9 (11)
C5—C4—H4A	119.8	H1—N3—H2	116.0 (16)
C3—C4—H4A	119.8	C13—N4—H4	117.5 (12)
C4—C5—C6	120.27 (11)	C13—N4—H5	122.8 (13)
C4—C5—H5A	119.9	H4—N4—H5	115.1 (17)
C6—C5—H5A	119.9	N3—C12—N2	118.40 (11)
C5—C6—C1	119.56 (11)	N3—C12—C13	122.85 (11)
C5—C6—C7	123.07 (11)	N2—C12—C13	118.68 (11)
C1—C6—C7	117.36 (10)	N4—C13—C14	122.73 (11)
O1—C7—C8	125.32 (11)	N4—C13—C12	119.69 (11)
O1—C7—C6	115.32 (10)	C14—C13—C12	117.57 (11)
C8—C7—C6	119.35 (10)	C13—C14—C15	121.28 (11)
C7—C8—C9	118.56 (10)	C13—C14—H14A	119.4
C7—C8—H8A	120.7	C15—C14—H14A	119.4
C9—C8—H8A	120.7	C16—C15—C14	119.68 (12)
N1—C9—C8	124.34 (10)	C16—C15—H15A	120.2
N1—C9—C10	117.26 (10)	C14—C15—H15A	120.2
C8—C9—C10	118.36 (10)	C15—C16—N2	119.11 (11)
O2—C10—O3	125.12 (11)	C15—C16—H16A	120.4
O2—C10—C9	117.50 (10)	N2—C16—H16A	120.4
			
C9—N1—C1—C2	177.07 (11)	C6—C7—C8—C9	−0.28 (16)
C9—N1—C1—C6	−0.38 (16)	C1—N1—C9—C8	3.32 (17)
N1—C1—C2—C3	−177.15 (11)	C1—N1—C9—C10	−174.09 (10)
C6—C1—C2—C3	0.41 (18)	C7—C8—C9—N1	−3.03 (18)
C1—C2—C3—C4	0.56 (19)	C7—C8—C9—C10	174.35 (10)
C2—C3—C4—C5	−0.68 (19)	N1—C9—C10—O2	−168.15 (11)
C3—C4—C5—C6	−0.19 (19)	C8—C9—C10—O2	14.27 (16)
C4—C5—C6—C1	1.15 (17)	N1—C9—C10—O3	14.14 (16)
C4—C5—C6—C7	179.93 (11)	C8—C9—C10—O3	−163.43 (11)
N1—C1—C6—C5	176.19 (11)	C16—N2—C12—N3	177.71 (11)
C2—C1—C6—C5	−1.25 (17)	C16—N2—C12—C13	0.46 (17)
N1—C1—C6—C7	−2.65 (16)	N3—C12—C13—N4	3.90 (17)
C2—C1—C6—C7	179.90 (11)	N2—C12—C13—N4	−178.98 (11)
C11—O1—C7—C8	−5.09 (17)	N3—C12—C13—C14	−176.96 (11)
C11—O1—C7—C6	176.10 (10)	N2—C12—C13—C14	0.16 (16)
C5—C6—C7—O1	2.97 (16)	N4—C13—C14—C15	178.25 (12)
C1—C6—C7—O1	−178.23 (10)	C12—C13—C14—C15	−0.86 (18)
C5—C6—C7—C8	−175.92 (11)	C13—C14—C15—C16	0.96 (19)
C1—C6—C7—C8	2.88 (16)	C14—C15—C16—N2	−0.34 (19)
O1—C7—C8—C9	−179.05 (11)	C12—N2—C16—C15	−0.37 (19)

### Hydrogen-bond geometry (Å, º)

**Table:**

*D*—H···*A*	*D*—H	H···*A*	*D*···*A*	*D*—H···*A*
N3—H2···O3	0.91 (2)	1.890 (19)	2.7670 (18)	162.8 (19)
N2—H3···N1	0.94 (2)	1.94 (3)	2.843 (2)	162.5 (19)
N3—H1···O3^i^	0.89 (2)	1.94 (2)	2.812 (2)	163.4 (16)
N4—H4···O2^ii^	0.893 (18)	1.978 (19)	2.8617 (15)	169.8 (17)
N4—H5···O2^i^	0.88 (2)	2.17 (3)	2.9419 (18)	146 (3)
C4—H4*A*···O2^iii^	0.95	2.45	3.3529 (15)	158

Symmetry codes: (i) −*x*+1, −*y*+2, −*z*+1; (ii) *x*, −*y*+3/2, *z*+1/2; (iii) *x*+1, −*y*+3/2, *z*+1/2.

## References

[bb1] Allen, F. H., Kennard, O., Watson, D. G., Brammer, L., Orpen, A. G. & Taylor, R. (1987). *J. Chem. Soc. Perkin Trans. 2*, pp. S1–19.

[bb2] Bernstein, J., Davis, R. E., Shimoni, L. & Chang, N.-L. (1995). *Angew. Chem. Int. Ed. Engl.* **34**, 1555–1573.

[bb3] Bruker (2009). *SADABS*, *APEX2* and *SAINT* Bruker AXS Inc., Madison, Wisconsin, USA.

[bb4] Cosier, J. & Glazer, A. M. (1986). *J. Appl. Cryst.* **19**, 105–107.

[bb5] Hemamalini, M. & Fun, H.-K. (2011). *Acta Cryst.* E**67**, o435–o436. 10.1107/S1600536811001541 PMC3051487 21523101

[bb6] Katritzky, A. R., Rees, C. W. & Scriven, E. F. V. (1996). In *Comprehensive Heterocyclic Chemistry II.* Oxford: Pergamon Press.

[bb7] Markees, D. G., Dewey, V. C. & Kidder, G. W. (1970). *J. Med. Chem.* **13**, 324–326. 10.1021/jm00296a048 5418519

[bb8] Morimoto, Y., Matsuda, F. & Shirahama, H. (1991). *Synlett*, **3**, 202–203.

[bb9] Pozharski, A. F., Soldatenkov, A. T. & Katritzky, A. R. (1997). In *Heterocycles in Life and Society.* New York: Wiley.

[bb10] Sheldrick, G. M. (2008). *Acta Cryst.* A**64**, 112–122. 10.1107/S0108767307043930 18156677

[bb11] Spek, A. L. (2009). *Acta Cryst.* D**65**, 148–155. 10.1107/S090744490804362X PMC2631630 19171970

